# Silk nanoparticles for the protection and delivery of guava leaf (*Psidium guajava* L.) extract for cosmetic industry, a new approach for an old herb

**DOI:** 10.1080/10717544.2023.2168793

**Published:** 2023-01-24

**Authors:** Duy Toan Pham, Doan Xuan Tien Nguyen, Ruby Lieu, Quoc Cuong Huynh, Ngoc Yen Nguyen, Tran Thi Bich Quyen, Van De Tran

**Affiliations:** a Department of Chemistry, College of Natural Sciences, Can Tho University, Can Tho, Vietnam; b Faculty of Commerce, Van Lang University, Ho Chi Minh City, Vietnam; c Faculty of Chemical Engineering, College of Engineering, Can Tho University, Can Tho, Vietnam; d Department of Health Organization and Management, Can Tho University of Medicine and Pharmacy, Can Tho, Vietnam

**Keywords:** Silk fibroin, nanoparticles, guava, *Psidium guajava* L., antioxidant, heat-protection

## Abstract

Guava (*Psidium guajava* L.) is a well-known plant containing high levels of natural antioxidants, the phenolic compounds, which have been employed in numerous cosmetic products. However, these molecules are unstable to oxidants, light, temperature, pH, water, and enzymatic activities. Therefore, to enhance their stability and preserve their antioxidant activity, this study investigated the silk fibroin nanoparticles (SFNs) ability to encapsulate, deliver, and heat-protect the phenolic compounds of the guava leaves ethanolic extract. Firstly, the guava ethanolic extract was produced by maceration, which possessed a total phenolic content of 312.6 mg GAE/g DPW and a high antioxidant activity (IC_50_ = 5.397 ± 0.618 µg/mL). Then, the extract loaded SFNs were manufactured by desolvation method, and the particles demonstrated appropriate sizes of 200–700 nm with narrow size distribution, spherical shape, silk-II crystalline structure, high drug entrapment efficiency of > 70% (dependent on the fibroin content), and a two-phase sustained drug release for at least 210 min. Using the 2,2-diphenyl-1-picrylhydrazyl (DPPH) assay, the antioxidant activity of the guava extract was well-preserved in the extract loaded SFNs. Finally, after being treated with high temperature of 70 **°**C for 24 h, the guava extract almost loses all of its antioxidant property (5 times decrement), whereas the extract loaded SFNs could retain the extract activity. Conclusively, the SFNs proved much potential to deliver and heat-protect the guava extract phenolic compounds, and preserve their antioxidant activity. Confirmed by this case, SFNs could be further explored in protecting other natural compounds from environmental factors.

## Graphical Abstract

**Figure:**
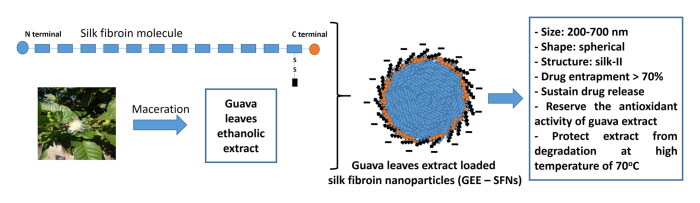

## 1. Introduction

Globally, the utilizations of natural herbal plants, especially their secondary metabolites known as phytochemicals, has been significantly increasing in cosmeceutical areas due to their safety, low extraction cost, and ease of scale-up (Mohammadinejad et al., 2019; Sukumaran et al., 2020). Plant phytochemicals consist of a broad variety of groups of compounds including phenolics/polyphenols, saponins, alkaloids, lignins, steroids, glycosides, and terpenoids (Soleimani et al., 2022). Among them, polyphenols play important roles in various beneficial biochemical and molecular processes in human body, namely anti-aging, chemoprevention (i.e. anti-mutagenicity and anti-carcinogenicity), and free radical scavenging (Cory et al., 2018). Therefore, polyphenols have been employed in numerous cosmetic products.

In the treasure of Vietnamese traditional plants, *Psidium guajava* L. (guava, Myrtaceae family), a well-known and common plant in Vietnam, in the ASEAN region, and in many tropical and subtropical areas (Shirur et al., 2013; Yusuf & Ocheje, 2019), is an ideal natural source of nontoxic polyphenols that act as antioxidant (Huda-Faujan et al., 2009) (i.e. chemicals that could eliminate free radicals, to prevent oxidative-related diseases like cancer, cardiovascular diseases, immune system, lung, and kidney disease (Houstis et al., 2006; Reiter, 1995)). The most abundant polyphenols in guava are myricetin and apigenin (Miean & Mohamed, 2001), ellagic acid, and anthocyanins (Misra & Seshadri, 1968). Numerous studies, both ethnopharmacologically and clinically, have evaluated and reported the outstanding antioxidant activity of guava, especially its leaves, in both in-vitro and in-vivo settings, in various applications fields such as food, medicine, and cosmetics (Chen & Yen, 2007; Tachakittirungrod et al., 2007; Gutiérrez et al., 2008; Nantitanon et al., 2010; Alvarez-Suarez et al., 2018; Nguyen et al., 2021; Tran et al., 2022). In fact, guava extracts have been employed in many commercial cosmetic products such as Guava seed oil (Chateau Cosmetics Botanical Beauty), GuavaStory products (cream, lotion, essence) (Guavaland), and Brazilian guava-leaf mosturizing kit (Natureza Cosmetics). Nevertheless, the guava-leaf polyphenol compounds, its main antioxidants, possess the general structures with aromatic rings consisted of unsaturated bonds, consequently making them vulnerable to various environmental factors such as light, heat, pH, water, and enzymes (Saénz et al., 2009). Moreover, clear associations between the polyphenols’ shelf-life stability and their antioxidant capacity have been reported (Hanuka Katz et al., 2020). Therefore, it is crucial to protect the guava phenolic compounds from these factors to increase their stability and efficacy in cosmetic products.

For this, one of the potential methods is to encapsulate the guava extract, especially its phenolic compounds, in a delivery systems such as nanoparticles. This technology is becoming increasingly important in the pharmaceutical, food, cosmetic, textile, chemical, biotechnology, and medicinal industries due to its potential for delivery and effective protection of bioactive compounds from harmful environmental agents (Microencapsulation, n.d.; Desai & Park, 2005). However, for the protection of polyphenols in herbal extracts, most studies focus on the synthetic polymeric nanoparticles (i.e. poly(lactide) (PLA), poly(lactide-co-glycolide) (PLGA)) and lipid-based nanoparticles (Pechanova et al., 2020; Yang et al., 2020), with insufficient emphasis on natural nanoparticles. To this end, a prospective material for exploring the nanoparticles potency in delivery herbal extract is silk fibroin, an adaptable protein that has been used for millennia and has been proposed as a biomaterial for encapsulation in food, cosmetics, and medicinal products (Pham & Tiyaboonchai, 2020; Pham et al., 2022). Silk fibroin, a protein commonly extracted from the cocoons of *Bombyx mori* silkworms, is a cheap, biocompatible, biodegradable, and nontoxic US FDA-approved polymer (Pham et al., 2020a; Pham & Tiyaboonchai, 2021). In addition, the extensive hydrogen bonding, amphiphilic nature, and high degree of crystallinity contribute to the great stability of silk fibroin (Altman et al., 2003). Silk fibroin nanoparticles (SFNs) have been successfully employed as a carrier for natural bioactive compounds such as resveratrol for the treatment of rat intestinal inflammation (Lozano-Pérez et al., 2014), alpha-mangostin for ulcerative colitis (Pham et al., 2020c), quercetin for antioxidant activity (Lozano-Pérez et al., 2017), and paclitaxel and curcumin for cancer treatment (Montalbán et al., 2018; Crivelli et al., 2019; Pham et al., 2019, 2020b). However, to the best of our knowledge, limited research have reported on the encapsulation of the whole herbal extracts, namely olive leaves extract (Bayçin et al., 2007; Altiok et al., 2008) and rosemary extract (Hcini et al., 2021), in SFNs. Furthermore, no study has investigated the SFNs ability to protect the phenolic compounds in the encapsulated extracts.

Therefore, to fill in the aforementioned knowledge gap, and to add more insights on the fibroin utilizations, this study developed and characterized the SFNs ability to encapsulate, protect, deliver, and activity-reserve the phenolic compounds of the guava leaves extract. To this end, the guava leaves were first extracted with ethanol by simple maceration method, followed by determining its total phenolic content and in-vitro antioxidant activity. Then, the extract was loaded in the SFNs, and the particles were characterized in terms of size, shape, morphology, structure, drug entrapment efficiency (EE%), drug loading capacity, drug release profiles, and antioxidant action. Finally, the heat-stability of both the guava extract and the SFNs containing guava extract was investigated.

## 2. Materials and methods

### 2.1. Materials

Guava leaves were collected in Can Tho City, Vietnam, in November 2021. The samples were identified based on the morphological description in the book ‘Plants of Vietnam’ (Hộ, 1999) and the voucher specimen were kept at the College of Natural Sciences, Can Tho University, Can Tho, Vietnam. The *Bombyx mori* silkworm, variety M45, was collected from Nam Dinh province, Vietnam. Other chemicals were of reagent grades or higher, including ascorbic acid (99%, Merck, Germany), gallic acid (Xilong, China), Folin-Ciocalteu (Merck, Germany), 2,2-Diphenyl-1-picrylhydrazyl (DPPH) (95%, Merck, Germany), Na_2_CO_3_ (99.8%, Xilong, China), ethanol 96% (Vietnam), and methanol 96% (Merck, Germany).

### 2.2. Guava leaves extraction

The collected fresh guava leaves were dried at ambient temperature and ground to fine powder. Then, 300 g of the powder was extracted with 1 L of ethanol using maceration method. After 24 h, the extract was collected and the remaining solid mass was extracted again with ethanol for another 2 times. Finally, all extracts were pooled, filtered, solvent evaporated by a rotavapor (Buchi, Switzerland) until semi-solid, and kept at 4 °C for further experiments.

### 2.3. Total phenolic content determination

The total phenolic content in the ethanolic extract of the guava leaves was determined by the Folin-Ciocalteu method, described by Singleton and Rossi, with some modifications (Singleton & Rossi, 1965). Briefly, 0.5 mL of the extract was mixed with 0.5 mL of distilled water, 0.5 mL of Folin-Ciocalteu reagent, and 0.5 mL of Na_2_CO_3_ solution (7.5% w/v). After 30 min of incubation at ambient temperature, the absorbance of the resulting blue-colored solution was UV-Vis spectroscopic measured at 765 nm. The standard curve was prepared by using the standard compound gallic acid, at different concentrations ranging from 0 to 10 µg/mL (y = 0.087x + 0.0016, R^2^ = 0.9916). The total phenolic content was calculated following Eq. (1) and was expressed as mg gallic acid equivalent/g dry powder weight (mg GAE/g DPW).

(1) $$\text{Total phenolic content }=\;\frac{C\times V\times k\times 10^{3}}{m}\text{ x }100$$

where C: concentrations of total phenolic content in the test solution (µg/mL); V: volume of the test solution (mL); k: dilution factor; m: weight of the dry powder (mg)

### 2.4. Silk fibroin extraction

Microwave-assisted hot extraction was used to extract fibroin from the silk cocoons, following the previous report (Pham et al., 2018). Ten grams (10 g) of silk cocoons were treated with 0.5% Na_2_CO_3_ solution at 100 °C for 1 h to eliminate sericin (i.e. the degumming process). After that, the product was cleaned with distilled water and dried naturally. Then, the degummed silk was dissolved in the CaCl_2_:H_2_O:Ca(NO_3_)_2_:EtOH mixture (mass ratio of 30:45:5:20), heated for 2 min in a microwave oven (900 W), and dialyzed against distilled water for 3–5 days at room temperature using a cellulose dialysis bag (10000 MWCO). To eliminate contaminants, the dialysis solution was centrifuged at 10000 rpm at 4 °C for 30 min. Finally, the silk fibroin solution was lyophilized and the freeze-dried fibroin was kept at 4 °C for further studies.

### 2.5. Preparation of blank SFNs and guava ethanolic-extract loaded SFNs (GEE–SFNs)

For the blank SFNs, three (03) formulas were prepared, at different initial fibroin concentrations of 1%, 2%, and 3% (w/v). For this, the freeze-dried fibroin powder was dissolved in 5 mL of distilled water to form the fibroin 1%/2%/3% solutions. Then, 2 mL of each fibroin solution was slowly added to 1 mL of ethanol and stirred at 4 °C for 24 h. Finally, the formed blank FMPs were centrifuged at 6000 rpm for 40 min, re-dispersed in distilled water, lyophilized, and stored at 4 °C for the next experiments (Pham et al., 2018).

For the GEE–SFNs, the process for fabricating 03 formulas, at the fibroin concentrations of 1%, 2%, and 3%, was performed similarly to the blank SFNs. However, 1 mL of ethanol was replaced with 1 mL of the guava ethanolic extract containing a total phenolic content of 2.5 mg/mL (Hcini et al., 2021).

### 2.6. Characterizations of blank SFNs and GEE–SFNs

The blank SFNs and GEE–SFNs were physico-chemically characterized in terms of the size and polydispersity index (PI) by dynamic light scattering (DLS) method, shape and morphology by field emission scanning electron microscopy (FESEM), structure by Fourier-transform infrared spectroscopy (FTIR), drug EE% and loading capacity by UV-Vis spectroscopy, and the drug release profile by shaker method.

For the size and PI, 150 mg of SFNs and GEE–SFNs samples were diluted in 5 mL of distilled water, homogenized using Misonix XL2020 Sonicator (USA), and the measurements were performed by Microtrac S3500 analyzer at 25 °C, at a fixed angle of 90° (Pham et al., 2018).

For the shape and morphology, the SFNs and GEE–SFNs dispersions were dropped onto the carbon-coated copper grid and the particles were dried naturally at ambient temperature. Then, the particles were coated with platinum and subjected to the FESEM sample holder. The operating procedure was then followed the manufacturer’s guideline.

The FTIR was used to determine potential structural changes in fibroin after loading with phenolic compounds of guava ethanolic extract. The spectra were recorded using an FTIR-4600 JASCO spectrometer with IQ accessory recognition. In 16 scans, measurements were taken in absorbance mode with a resolution of 1 cm^−1^ and a spectral range of 4000–400 cm^−1^, utilizing Cosine apodization and a TGS detector. The investigation concentrated on the 1700–1400 cm^−1^ and 3200–2800 cm^−1^ regions, which provide the greatest information on the FTIR spectra of fibroin and the SFNs.

The EE% and the loading capacity of the total phenolic content in the GEE–SFNs were measured by indirect method using the Folin-Ciocalteu reagent. Briefly, after the GEE–SFNs were formed, the particles were collected by centrifugation, and the supernatant (0.5 mL) containing the unloaded extract was mixed with 0.5 mL of distilled water, 0.5 mL of Folin-Ciocalteu reagent, and 0.5 mL of Na_2_CO_3_ solution (7.5%). After 30 min of incubation, the absorbance of the resulting blue-colored solution was measured at 765 nm to calculate the unloaded phenolic content, and the loaded phenolic content in SFNs was then determined. The EE% and the total phenolic loading (TPL%) were calculated using Eqs. (2) and (3), respectively.

(2) $$\text{EE\% }=\frac{\text{Amount of total phenolic content in SFNs}}{\text{The initial amount of total phenolic content}}\times 100\%$$

(3) $$\mathrm{TPL}\%=\frac{\text{Amount of total phenolic content in SFNs}}{\text{The total amount of particles}}\times 100\%$$

For the drug release profile, the total phenolic release was determined by shaker method at 37 ± 0.5 °C. Briefly, 10 mg of lyophilized GEE–SFNs were dispersed in 50 mL of the phosphate buffered saline (pH 7.4) and shaken at 200 rpm for 210 min. At each time point, 1 mL of sample was withdrawn and the same amount of buffer was re-filled. The sample was centrifuged at 18000 rpm for 5 min (Pham et al., 2018), and the total phenolic release from GEE–SFNs in the supernatant was reacted with Folin-Ciocalteu reagent and determined by UV-Vis spectrophotometer at 765 nm. Finally, the cumulative percentages of total phenolic release were calculated following Eq. (4).

(4) $$\text{\% Cumulative release }=\frac{C_{t}V_{0}+V\sum_{1}^{t-1}C_{i}}{M_{0}-\sum_{1}^{t-1}M_{i}}x\;100\%$$

Where C_t_, C_i_: concentrations of released total phenolic at the time point t and i; V_0_: total volume of dissolution buffer (50 mL); V: withdrawal sample volume at each time point (1 mL); M_0_: initial amount of total phenolic content; M_i_: total amount of total phenolic withdrawal at the time point i

### 2.7. In-vitro antioxidant DPPH assay

The radical scavenging activities of the guava ethanolic extract, the blank SFNs, and the GEE–SFNs were analyzed following the method described by Tailor and Goyal with some modifications (Tailor & Goyal, 2014). For the extract, 40 µL of DPPH in methanol (1 mg/mL) was mixed with 960 µL of the extracts at different concentrations (0, 2, 4, 6, 8, and 10 µg/mL). The scavenging activities of the samples and the standards (ascorbic acid, 1–7 µg/mL in methanol) were measured at 517 nm after 30 min of reaction in the dark at 25 °C. The radical scavenging activity was then calculated by Eq. (5), and the sample IC_50_ was determined based on plotted calibration curves between the scavenging activity and the sample concentration.

(5) $$\mathrm{DPPH}\;\mathrm{scavenging}\;\mathrm{effect}\;(\%)\;=\;\frac{{\mathrm{Abs}}_{1}-\;{\mathrm{Abs}}_{2}}{{\mathrm{Abs}}_{1}}\text{ x }100$$

where Abs_1_ and Abs_2_ represent the absorbance values of the control (samples with no extracts) and the test samples, respectively.

For the GEE–SFNs, the particles aqueous dispersions (960 µL) were mixed with 40 µL of DPPH in methanol (1 mg/mL) to initiate the reaction. To determine whether the guava extract scavenging activity still retained after the extract was loaded in SFNs, the radical scavenging activity was measured at different incubation time of 30 min, 90 min, and 180 min. Then, the samples were centrifuged at 2000 rpm for 2 min to limit the influence of SFNs, and the supernatant was evaluated by measuring the absorbance at 517 nm. The particle scavenging activity was calculated by Eq. (5), with the blank SFNs as control.

### 2.8. Effect of temperature on the antioxidant activity of the extract and the GEE–SFNs

To evaluate the SFNs ability to heat-protect the phenolic compounds antioxidant activity of the guava leaves extract, both the pure extract and the GEE–SFNs were kept at 70 °C for 24 h, and being subjected to the DPPH assay (Sec. 2.7). Briefly, 960 µL of the extract or the GEE–SFNs aqueous dispersion, at a total phenolic content corresponding to the extract IC_50_ values, were mixed with 40 µL of DPPH in methanol (1 mg/mL). After 30 min of reaction at 25 °C  in the dark, the scavenging activities of the samples were evaluated by measuring the absorbance at 517 nm.

### 2.9. Statistical analysis

The quantitative experiments were conducted in triplicate to confirm the results, and the data were reported as mean ± SD (standard deviation). Student’s t-test and one-way analysis of variance (ANOVA) were utilized for statistical purposes, where necessary, with the p value of <.05 for significant comparisons.

## 3. Results and discussion

The cosmeceutical products containing guava extracts have been commercially available worldwide, mainly for the purpose of antioxidant. However, most, if not all, products employ the pure extracts in the formulation, which could hinder the guava polyphenol efficacy due to environmental degradation. Therefore, this study investigates the SFNs ability to encapsulate and deliver the phenolic compounds in the guava leaves extract, to reserve the extract antioxidant activity, and to protect the loaded compounds from high temperature. To this end, the guava leaves were extracted and evaluated its total phenolic content, then, the extract was loaded into the SFNs and the particles were fully characterized. Finally, the pure extract and the extract loaded SFNs were investigated their antioxidant activities at the normal condition as well as after high-heat treatments.

### 3.1. Guava leaves extraction

The guava leaves extraction efficiency was 12.82 ± 0.91%, and the extract possessed a dark-green color with a moisture content of 6.38 ± 0.04%, which was appropriate for further experiments. The total phenolic content in the GEE was 312.6 ± 0.14 mg GAE/g DPW. Our result was higher than that of the previous studies, which showed that the total phenolic content in the guava leaves were 53.04 ± 14.85 mg GAE/g fresh weight (Iamjud et al., 2014) and ∼110 mg GAE/g DPW (Seo et al., 2014). This could be due to the differences of the guava leaves from different region, soil and environmental conditions, and cultivation time, which result in different bioactive compounds contents.

### 3.2. SFNs and GEE–SFNs formulations

**Figure 1.**
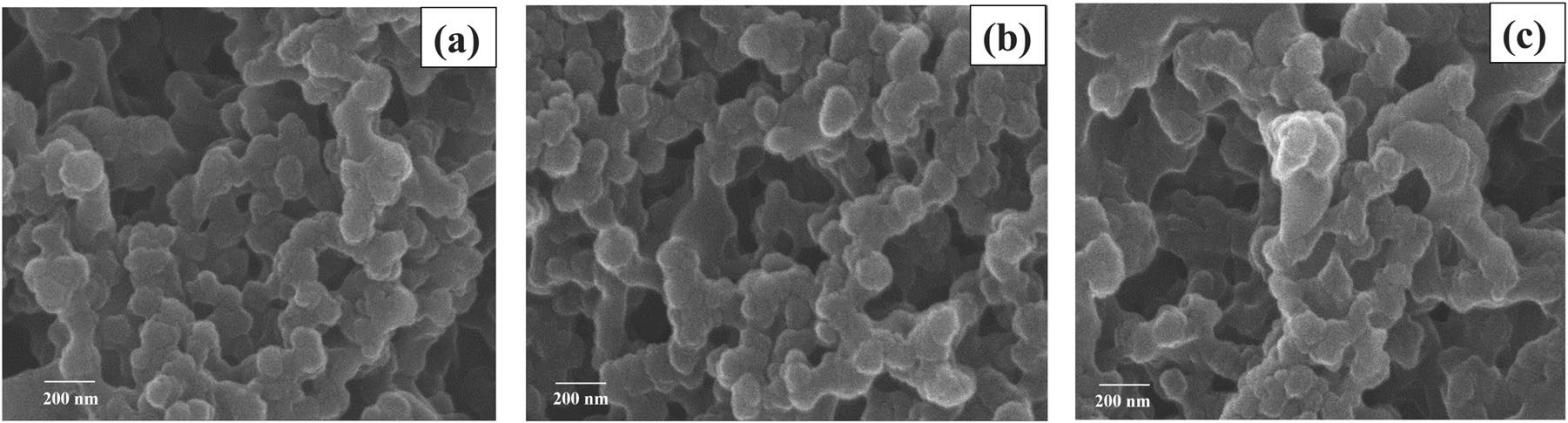
Scanning electron microscopic (SEM) images of the guava ethanolic-extract loaded silk fibroin nanoparticles (GEE–SFNs) at different fibroin concentrations of (a) 1%, (b) 2%, and (c) 3%.

**Figure 2.**
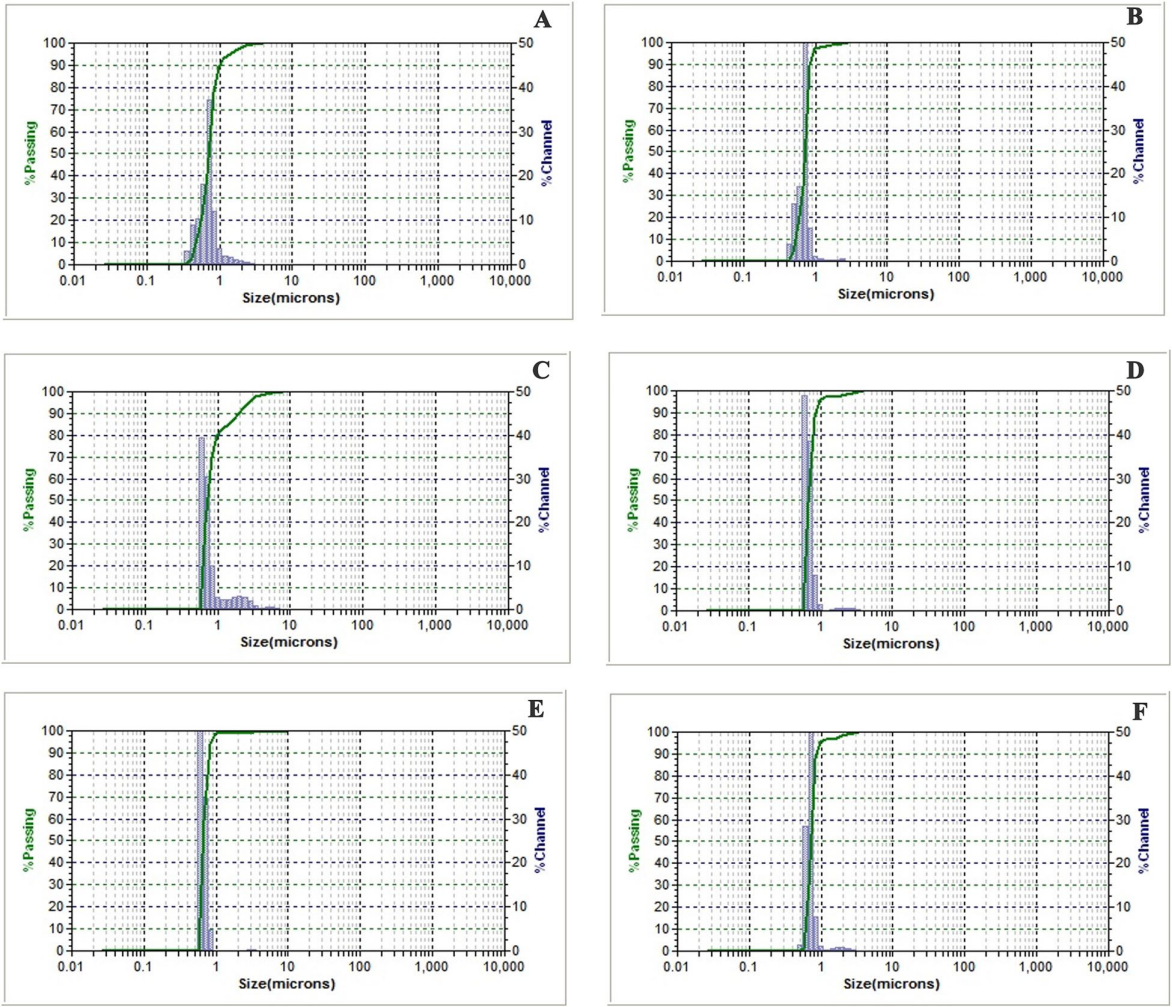
Particle sizes (µm) and size distributions by passing (%), measured by dynamic light scattering (DLS) method, of (A) blank silk fibroin nanoparticles (SFNs)—1% fibroin, (B) blank SFNs—2% fibroin, (C) blank SFNs—3% fibroin, (D) guava ethanolic-extract loaded SFNs (GEE–SFNs)—1% fibroin, (E) GEE–SFNs—2% fibroin, and (F) GEE–SFNs—3% fibroin..

Three blank SFNs and three GEE–SFNs were manufactured, at three different fibroin concentration of 1%, 2%, and 3%. The SEM images (Figure 1) revealed that all formulas possess a pseudo-spherical morphology of about 200 nm in size, which was slightly smaller than the hydrodynamic sizes obtained by the DLS measurements (Table 1 and Figure 2). This may be due to agglomeration effects, since the signals obtained from the DLS might not be from individual particles but from a cluster of aggregated particles. This result was similar to the previous studies describing that DLS could not differentiate between primary particles and their agglomerates (Lozano-Pérez et al., 2014; 2015; Hcini et al., 2021). Furthermore, comparing the sizes of the blank SFNs and the GEE–SFNs, the extract loaded particles showed bigger sizes, indicating the effects of drug encapsulation on the SFNs properties, as reported previously (Chomchalao et al., 2020). On the other hand, the fibroin concentrations did not significantly affect the SFNs sizes. Lastly, all formulas possess narrow size distributions with PI < 0.3.

**Table 1.** Particles sizes and polydispersity indexes (PI) of the blank silk fibroin nanoparticles (SFNs) and the guava ethanolic-extract loaded SFNs (GEE–SFNs).

	Fibroin concentration	Size (nm)	PI
Blank SFNs	1%	535 ± 42	0.100 ± 0.031
	2%	645 ± 80	0.030 ± 0.004
	3%	618 ± 11	0.200 ± 0.023
GEE–SFNs	1%	732 ± 14	0.020 ± 0.001
	2%	746 ± 12	0.010 ± 0.003
	3%	758 ± 37	0.010 ± 0.002

The values are expressed in terms of mean ± SD (*n* = 3).

Regarding the EE% and TPL% of the GEE–SFNs, Table 2 demonstrates that the EE% significantly increased from 70% to 85% with increasing fibroin concentration from 1% to 3%. On the other hand, the TPL% decreased proportionally to the fibroin concentrations. This could be explained by the saturation effect. Since the fibroin molecules in the SFNs interact directly with the phenolic compounds in the GEE, an increase in the fibroin concentration (i.e. the fibroin amount) yielded more and effective interactions, consequently, the EE% was enhanced. However, when comparing to the whole particles mass, the more the fibroin content (i.e. more SFNs), the less the TPL%. This indicates that the SFNs ability to encapsulate the phenolic compounds in the GEE has reached its saturation state at a fibroin concentration of 2%.

**Table 2.** The polyphenol entrapment efficiency (EE%) and total phenolic loading (TPL%) of the guava ethanolic-extract loaded silk fibroin nanoparticles (GEE–SFNs) at different fibroin concentration of 1%, 2%, and 3%.

	Fibroin concentration	EE%	TPL%
GEE–SFNs	1%	68.053 ± 0.023	0.280 ± 0.001
	2%	79.291 ± 0.002	0.100 ± 0.003
	3%	85.667 ± 0.002	0.090 ± 0.002

The values are expressed in terms of mean ± SD (*n* = 3).

**Figure 3.**
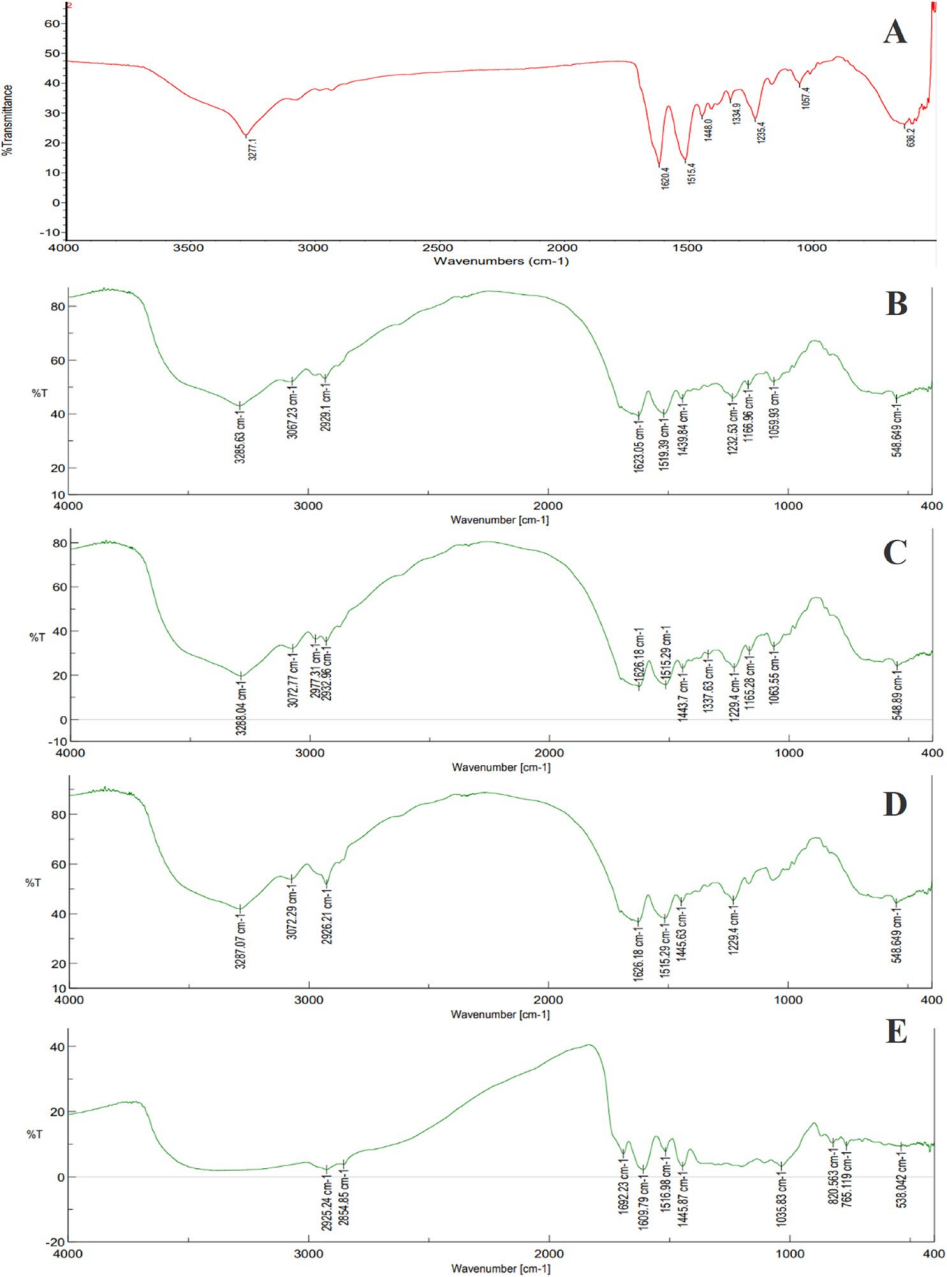
Fourier-transform infrared spectroscopy (FTIR) spectra of (A) blank silk fibroin nanoparticles (SFNs), (B) guava ethanolic-extract loaded SFNs (GEE–SFNs)—1% fibroin, (C) GEE–SFNs—2% fibroin, (D) GEE–SFNs—3% fibroin, and (E) guava ethanolic extract.

Regarding the particle structures, the FTIR spectra of the blank SFNs, the GEE–SFNs, and the GEE are shown in Figure 3. Firstly, both the blank SFNs and the GEE–SFNs possessed similar silk-II-crystalline fibroin characterized peaks at 1623–1626 cm^−1^ (C = O stretching of the fibroin amide-I area) and at 1519–1515 cm^−1^ (N-H bending of fibroin amide-II area) (Hu et al., 2006; Zhang et al., 2007; Lozano-Pérez et al., 2014; Pham et al., 2018). This suggests that the formulation process has altered the raw fibroin silk-I amorphous structure to silk-II crystalline structure in the SFNs. Secondly, the FTIR of GEE–SFNs showed some characterized signals of the GEE itself, with strong and broad absorption bands in the range of 3000–3700 cm^−1^ (O-H stretching vibrations of alcoholic/phenolic groups, and hydrogen bonding (Falcão & Araújo, 2013)) and small bands at 2854 and 2925 cm^−1^ (C-H stretching vibrations of the CH_2_ and -CH groups). This confirms the successful encapsulation of the GEE in the SFNs. Conclusively, the guava leaves extract was loaded in the nanoparticles, and the extract phenolic compounds did not alter the SFNs structures.

**Figure 4.**
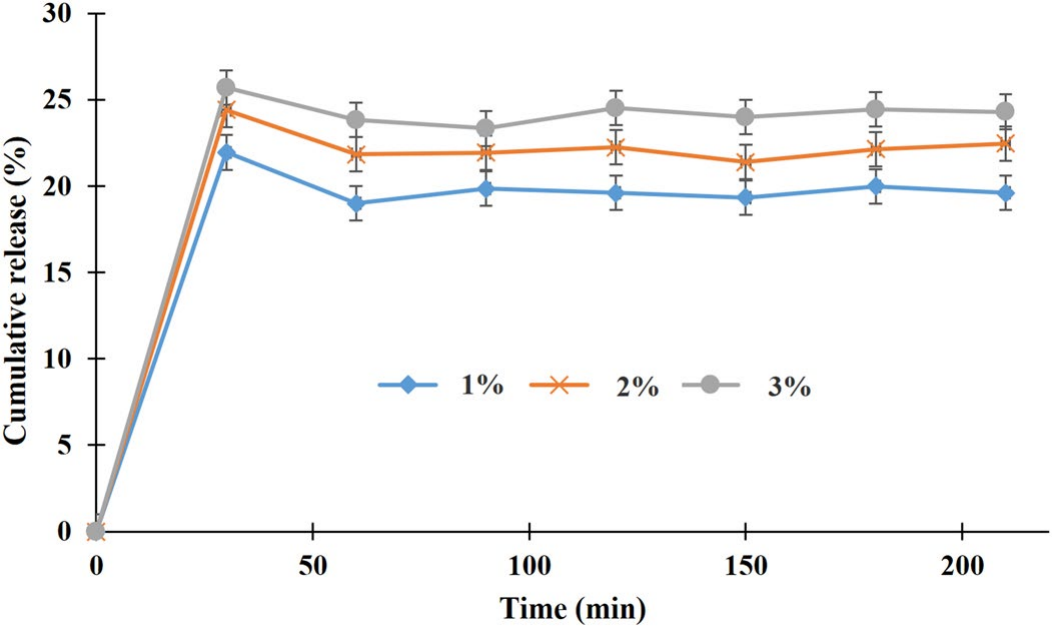
Phenolic compound release profiles of the guava ethanolic-extract loaded silk fibroin nanoparticles (GEE–SFNs), at different fibroin concentrations of 1%, 2%, and 3%, in phosphate buffer saline at pH 7.4 (*n* = 3).

In terms of the phenolic-compound release pattern of the GEE–SFNs (Figure 4), the release process can be divided into two main stages. For the first stage, during the initial 30 min, the percentage of released phenolic-compounds significantly increased and reached the highest level of 21.96%, 24.42%, and 25.71% for formulas with fibroin concentrations of 1%, 2%, and 3%, respectively. For the second stage, from 30 min to 210 min, a sustained release profile was observed. This phenomenon was in accordance with the previous report that investigated the release rate of quercetin loaded SFNs (Lozano-Pérez et al., 2017). Since the guava leaves ethanolic extract contains various polyphenol compounds, it is certain that some of them adsorbed on the surface of the particles, whereas other homogenously dispersed in the particles core. Thus, the compounds on the surfaces would release faster than the others. Another notable issue is that the release amount of the 3%-fibroin-formula was significantly higher than the formulas with 1% and 2% fibroin. This was in correspondent with the EE% results, and thus, suggests that the more the polyphenol interacts with the fibroin, the higher its release rate.

### 3.3. In-vitro antioxidant DPPH assay

Regarding the antioxidant activities, the DPPH free radical scavenging capacity of the guava leaves extract, the blank SFNs, and the GEE–SFNs, were evaluated. To this end, the extract antioxidant activity was directly proportional to its phenolic contents, re-confirming the extract activity is mainly due to its phenolic compounds. The extract possessed a maximum DPPH free radical scavenging efficiency of 95.784% at the phenolic concentration of 10 µg/mL, with a concentration-action correlation equation of y = 9.5489x − 1.5327 (R^2^ = 0.9961), and an IC_50_ of 5.397 ± 0.618 µg/mL. This result indicates that the guava leaves extract shows relatively high antioxidant action, as compared with the reference vitamin C (IC_50_ = 4.209 ± 0.571 µg/mL). Our extract demonstrates higher activity than that of the report of Manikandan et al. (2017), which showed an IC_50_ value of 18.01 µg/mL (Manikandan et al., 2017). This difference could be attributed to each region’s diverse soil and environmental conditions, which result in different biological activities. Those antioxidant properties were associated with the extract phenolic compounds such as protocatechuic acid, ferulic acid, quercetin, guavin B, ascorbic acid, gallic acid, and caffeic acid (Jiménez-Escrig et al., 2001; Thaipong et al., 2005).

Next, the DPPH free radical scavenging capacity of the blank SFNs was investigated (Table 3). The results showed that all formulas, at different fibroin concentrations of 1%, 2%, and 3%, possessed a low radical scavenging activity of < 5%. This could be due to the presence of tyrosine residues in the silk fibroin sequence (Hcini et al., 2021).

**Table 3.** The 2,2-Diphenyl-1-picrylhydrazyl (DPPH) scavenging activity (%) of the blank silk fibroin nanoparticles (SFNs) and the guava ethanolic-extract loaded SFNs (GEE–SFNs) at different fibroin concentration of 1%, 2%, and 3%, and at different testing time of 30 min, 90 min, and 180 min.

	Fibroin concentration	Testing time		
		30 min	90 min	180 min
Blank SFNs	1%	5.751 ± 0.063%	5.762 ± 0.053%	6.105 ± 0.071%
	2%	4.118 ± 0.034%	5.429 ± 0.050%	5.554 ± 0.053%
	3%	4.753 ± 0.009%	5.003 ± 0.017%	5.658 ± 0.043%
GEE–SFNs	1%	36.283 ± 0.013%	46.136 ± 0.183%	54.595 ± 0.004%
	2%	33.664 ± 0.132%	39.251 ± 0.021%	52.595 ± 0.232%
	3%	37.279 ± 0.382%	47.873 ± 0.182%	58.142 ± 0.029%

The values are expressed in terms of mean ± SD (*n* = 3).

Finally, the DPPH free radical scavenging ability of the GEE–SFNs was evaluated (Table 3). The findings revealed that the particle could well-preserve the antioxidant efficiency of the encapsulated guava leaves extract. Interestingly, the GEE–SFNs scavenging actions gradually increased over time, from 30% at 30-min incubation to 50% at 180-min incubation with DPPH. Compared with the pure guava leaves extract, the GEE–SFNs efficacy was weaker at the 30-min incubation, however, this efficacy increased to be nearly equal to the extract after 180 min of incubation. The results indicate that the antioxidant effectiveness of the GEE–SFNs is dependent on the release of phenolic compounds from the particles, allowing for further investigation into the control of drug release for the most effective action at the target.

### 3.4. Effect of temperature on the antioxidant activity of the extract and the GEE–SFNs

To investigate the SFNs ability to protect the phenolic compounds in the guava leaves extract from the external environmental factors such as high temperature, both the pure extract and the GEE–SFNs were subjected to a high heat of 70 °C for 24 h. This temperature has been proven to cause degradation in most polyphenol structures. Then, the GEE–SFNs were re-determined their physical properties as described in Sec. 2.6. For this, all formulas parameters including the size, PI, morphology, and structure were preserved (i.e. the differences between the temperature-induced particles and the initial particles were less than 10%) (data not shown). Afterwards, the temperature-induced extract and GEE–SFNs, at a concentration corresponding to the IC_50_ value, were tested their antioxidant activity by DPPH assay. The results revealed that the antioxidant activities of both the extract and the GEE–SFNs were affected by temperature through the reduction of DPPH free radical scavenging performance (Table 4). Interestingly, the temperature-induced GEE–SFNs demonstrated significantly less reduction in antioxidant activity (i.e. 1.5 times) compared to the temperature-induced extract, with nearly 5 times decrement. Since high temperature treatment (i.e. 70 °C) has been proven to statistically degrade the guava leaf polyphenol content, namely gallic acid, catechin, epicatechin, quercetin, and chlorogenic acid (Nguyen et al., 2022), our results prove that the SFNs played an important role in encapsulating and protecting the phenolic compounds of guava leaves extract against high temperature.

**Table 4.** The 2,2-Diphenyl-1-picrylhydrazyl (DPPH) scavenging activity (%) of the guava leaves extract and the guava ethanolic-extract loaded silk fibroin nanoparticles (GEE–SFNs), before and after being treated at high temperature of 70 °C for 24 h, at different fibroin concentration of 1%, 2%, and 3%.

	Fibroin concentration	DPPH scavenging activity (%)	
		Before heat treatment	After heat treatment
GEE–SFNs	1%	36.283 ± 0.013%	24.585 ± 0.024*
	2%	33.664 ± 0.132%	26.806 ± 0.012*
	3%	37.279 ± 0.382%	28.789 ± 0.063*
Guava leaves ethanolic extract		50.906 ± 0.479%	11.863 ± 0.134*

The values are expressed in terms of mean ± SD (*n* = 3).

*Significant differences between values in the same row (*p* < .05).

## 4. Conclusions

This study investigated the ability of silk fibroin nanoparticles to encapsulate and heat-protect the phenolic compounds of the guava (*Psidium guajava* L.) leaves ethanolic extract, as a potential natural antioxidants for cosmeceuticals areas. The particles were successfully formulated with appropriate size (∼0.2–0.7 µm), narrow size distribution (PI < 0.3), spherical shape, silk-II-crystalline structure, high drug entrapment efficiency (EE% > 70%), and two-phase controlled drug release profile. Moreover, using the DPPH assay, the antioxidant activity of the extract in the particles was conserved and could be controlled in a time-dependent manner. Lastly, the particles could significantly protect the extract from degradation at high temperature of 70 °C for 24 h, and partly reserve its antioxidant effect. In summary, the silk fibroin nanoparticles are a prospective delivery system for the phenolic compounds of the guava extract. Proven by this case, the potency of these particles in protecting various natural compounds from environmental factors should be further explored.
